# Critical care nurses’ knowledge and attitudes and their perspectives toward promoting advance directives and end-of-life care

**DOI:** 10.1186/s12912-022-01066-y

**Published:** 2022-10-13

**Authors:** Mu-Hsing Ho, Hsiao-Chi Liu, Jee Young Joo, Jung Jae Lee, Megan F. Liu

**Affiliations:** 1 School of Nursing, LKS Faculty of Medicine, The University of Hong Kong, 5/F, Academic Building 3 Sassoon Road, Pokfulam, Hong Kong, Hong Kong; 2 Department of Nursing, Far Eastern Memorial Hospital, 21 Nanya S. Rd., Sec. 2, Banciao Dist., New Taipei City, 220 Taiwan; 3 College of Nursing, Gachon University, 191 Hambakmoero, Yeonsu-gu, Incheon, Korea; 4 School of Gerontology and Long-Term Care, College of Nursing, Taipei Medical University, 250 Wu-Hsing Street, Taipei, 11031 Taiwan

**Keywords:** Advance directive, Critical care nurse, End-of-life, Knowledge, Attitude

## Abstract

**Background:**

End-of-life care can be a difficult and challenging process for critical care nurses in intensive care units (ICUs) due to the care plan shifts from providing life-sustaining measures to end-of-life care. The aims of this study were to assess critical care nurses' perceived knowledge and attitudes toward end-of-life care, as well as their perspectives on promoting advance directives and the associated factors.

**Methods:**

A cross-sectional study was undertaken in an acute major metropolitan medical center in northern Taiwan between February and March 2020, and 250 critical care nurses were invited to participate in the study. Data on demographics, self-perceived knowledge of end-of-life care, attitudes toward end-of-life care, and perspectives of promoting advance directives were collected. A multiple linear regression model with stepwise selection was used to identify factors associated with their perspectives of promoting advance directives.

**Results:**

The law related to end-of-life care was rated as the least familiar part of the self-perceived end-of-life care knowledge, while ‘I have sufficient knowledge to care for patients who have accepted end-of-life care’ was the lowest level of agreement in attitude scores among critical care nurses. Increased levels of perceived knowledge (*β* = 0.134; *p* = 0.045) and attitudes (*β* = 0.423; *p* < 0.001) toward end-of-life care were associated with the perspectives of promoting advance directives. Nurses who worked in cardiac (*β* = -0.234; *p* < 0.001) and respiratory ICUs (*β* = -0.135; *p* = 0.024) had less motivation to promote advance directives (*F* = 16.943; *p* < 0.001).

**Conclusion:**

Given their important contributions to ICU care services, appropriate and meaningful support is required to optimize critical care nurses' involvement in end-of-life care. This study demonstrated a significant impact on perspectives of promoting advance directives of critical care nurse participants. Findings from this study can inform the design of effective nurse support programs to enhance the promotion of advance directives in intensive care settings.

## Introduction

In Taiwan, the *Hospice Palliative Care Act* was enacted into law in 2000, providing patients suffering from a terminal illness who were certified by two physicians the authority to sign advance directives and do not resuscitate (DNR) orders [1]. A directive can be made in one of two ways “*(1) it can be signed by a competent terminally ill patient or (2) it can be signed by a surrogate decision-maker, most commonly a close family member when a patient is unable to make such a decision for themselves due to dementia, delirium, or other types of cognitive impairment*” [1]. Advance directives are regarded as “a declaration, typically in writing, in which a person stated when mentally competent the type of health care he would desire to have at a future time when he is no longer capable [2].” Advance directives can assist healthcare personnel in respecting and following the preferred treatment of patients [3]. Before deciding on a DNR directive, the physician must consult with the patients and their family members. Initially, this used to exclusively apply to cancer patients in terminal condition, allowing them to receive hospice palliative treatment and make a DNR decision. Later modifications in 2009 increased insurance coverage of hospice and palliative care treatments to include non-malignant terminal conditions such as heart failure, chronic renal failure, liver cirrhosis and severe dementia. One of the most often used advance directives in end-of-life care is that when a person refuses to accept cardiopulmonary resuscitation (CPR) in the case of cardiac or respiratory arrest [1].

End-of-life care can be a difficult and challenging process in an intensive care unit (ICU) because many terminal illnesses involve withholding or withdrawing life-sustaining therapies, and in such situations, the role of critical care nurses shifts from providing life-sustaining measures to end-of-life care [4, 5]. Critical care nurses face these terminal illnesses and provide direct care to critically ill patients in the ICU, and they are in the best position to promote advance directives and provide end-of-life care. However, a study in Taiwan discovered that critical care nurses have significantly lower knowledge of and attitudes toward advance directives than ICU physicians. Also, a disagreement about end-of-life decision-making between ICU physician and nurses was found regarding who initiated conversation about end-of-life care discussion [6]. Insufficient knowledge and low awareness of advance directives are likely to be the reasons that result in challenges for critical care nurses to provide end-of-life care in ICUs [7]. Given the importance of the involvement of critical care nurses in end-of-life care such as decision-making and promoting advance directives, more strategies for practice development to prepare and support nurses in providing end-of-life care in intensive care settings are needed [6, 8]. Understanding the perceived knowledge of and attitudes toward end-of-life care can inform further development of strategies to support nurses in providing end-of-life care and promoting advance directives. Thus, this study aimed to assess (1) critical care nurses' self-perceived end-of-life care knowledge and attitudes toward terminal patients, as well as their perspectives of promoting advance directives; and (2) the factors associated with critical care nurses’ perspectives of promoting advance directives.

## Methods

### Research design and setting

A cross-sectional survey was conducted among critical care nurses between February and March 2020. The research site was a major acute-care metropolitan medical center with approximately 120 ICU beds located in northern Taiwan.

### Participants

Critical care nurses who met the selection criteria were invited to participate in this study. The inclusion criteria were a status as a registered nurse working in an ICU and agreed to participate in this study. Nurses who were receiving a temporary ICU training in the ICU were excluded. Critical care nurses who worked in a pediatric or neonatal ICU or the emergency room were also excluded, given the nature of nursing practice and workload, admitted patients, and the equipment/medications used in between adult ICU, pediatric or neonatal ICU, and the emergency room were different in Taiwan. G* power vers. 3.0.10 software was used to estimate the sample size [9]. The statistical test and model settings for the sample size estimation were as follow: *F* test as the test family; linear multiple regression: fixed model; *R*^*2*^ deviation from zero as the statistical test with a prior power analysis (given *α*, power, and effect size); and parameter settings (*α* = 0.05, 1–*β* = 0.95. *f*^*2*^ effect size = 0.08, and number of predictors = 3). Parameter settings were established according to a previous study on perspectives of promoting advance directives as an outcome [10]. An estimated sample size of 219 participants was considered sufficient.

### Ethical approval

The study protocol was reviewed and approved by the Hospital Institutional Review Board of the research site. Ethical approval was granted with approval no.: 108152-F. Written consent was obtained prior to the commencement of data collection. Subjects who were invited to participate could choose to discontinue their participation at any time.

### Data collection

A survey was developed based on a literature review and expert input. The tool was comprised of four parts that had to be completed, namely demographics, self-perceived knowledge of end-of-life care, attitudes toward end-of-life care, and perspectives of promoting advance directives. The survey was distributed by a research assistant to potential participants in person who agreed to join the study.

#### Demographic characteristics

Information of critical care nurses we collected included gender, age, educational level, years working as an ICU nurse, type of their workplace ICU, and continuing education in end-of-life care per year (hours).

#### Self-perceived end-of-life care knowledge of terminal patients

In total, 23 questions and a five-point Likert scale were used. Positively keyed questions were assigned 5 as “very familiar”, 4 as “familiar”, 3 as “neither familiar nor unfamiliar”, 2 as “unfamiliar”, or 1 as “very unfamiliar”. The higher the score, the higher the degree of self-perceived end-of-life care knowledge of critical care nurses, and the higher the tendency that critical care nurses would provide end-of-life care. This scale was developed to examine self-perceived end-of-life care knowledge towards terminal patients among critical care nurses. The content validity and the internal consistency (Cronbach’s alpha = 0.94) were established [11].

#### End-of-life care attitudes towards terminal patients

In total, 17 questions and a five-point Likert scale were adopted, with 5 as “extremely agree”, 4 as “agree”, 3 as “neither agree nor disagree”, 2 as “disagree”, or 1 as “extremely disagree”. The higher the score, the more positive care attitudes towards terminal patients from critical care nurses, and the higher the tendency that the critical care nurses would provide end-of-life care. This scale aimed to assess end-of-life care attitudes among critical care nurses towards terminal patients. The content validity and the internal consistency (Cronbach’s alpha = 0.85) were confirmed [11].

#### Perspectives of promoting advance directives

Perspectives of promoting advance directives scale were adopted from Hsieh et al. (2010) who developed a test for Taiwanese nursing staff. In total, 24 questions with a five-point Likert scale were used, with 1 as “extremely disagree”, 2 as “disagree”, 3 as “neither agree nor disagree”, 4 as “agree”, and 5 as “extremely agree”. Negatively keyed questions were inversely scored when summary scores were computed. The total score of the questionnaire ranged 24 ~ 120 points. The higher the score, the more-positive attitudes nursing staff had towards promoting advance directives. A lower score indicated that there would be challenges or negative attitudes towards discussing advance directives between nursing staff and patients. The content validity and internal consistency (Cronbach’s alpha = 0.80) were confirmed [10].

## Data analysis

All data were entered into SPSS© vers. 25.0 for analysis (IBM SPSS Statistics for Windows, vers. 25.0. IBM, Armonk, NY, USA). Descriptive analyses, including the mean, standard deviation (SD), and frequency distributions were used to summarize data relating to demographics, self-perceived knowledge of end-of-life care, attitudes toward end-of-life care, and perspectives of promoting advance directives. Multiple imputation was employed to handle missing values. A multiple linear regression model with stepwise selection was used to identify factors associated with perspectives of promoting advance directives. All variables including demographics were entered in the regression model, and significant variables were selected and included in the final model. The variance inflation factor (VIF) was also examined to discover potential multicollinearity issues between variables. Statistical significance for all tests was set to *p* < 0.05.

## Results

### Participants

In total, 250 responses were received. Of these, 88.4% (*n* = 220) of subjects were female, and 76.0% (*n* = 341) were aged 20 ~ 30 years (Table 1). Ninety-two percent (*n* = 231) of participants had an undergraduate education level, and more than half (*n* = 148; 59.2%) had been an ICU nurse for 1 ~ 5 years. Most (*n* = 76; 30.4%) participants worked in a medical ICU, followed by surgical ICUs (*n* = 57; 22.8%) and cardiac ICUs (*n* = 54; 21.6%). Almost all participants (*n* = 244; 98.0%) received ≤ 5 h of continuing education in end-of-life care per year.

**Table 1:** Demographic characteristics of participants (*N* = 250)

Item	*n* (%)
Gender (*n* = 249)	
Male	29 (11.6)
Female	220 (88.4)
Age group, years	
20 ~ 30	190 (76.0)
31 ~ 40	45 (18.0)
41 ~ 50	15 (6.0)
Educational level (*n* = 249)	
Tertiary education	12 (4.8)
Undergraduate	231 (92.4)
Master's degree	6 (2.4)
Year working as an ICU nurse	
1 ~ 5	148 (59.2)
6 ~ 10	54 (21.6)
11 ~ 15	27 (10.8)
> 15	21 (8.4)
Type of ICU	
Medical	76 (30.4)
Surgical	57 (22.8)
Cardiac	54 (21.6)
Neurologic	27 (10.8)
Respiratory	36 (14.4)
Continuing education in EoL care per year (h) (*n* = 249)	
0	45 (18.1)
0.5	80 (32.1)
≦5	119 (47.8)
6 ~ 10	4 (1.6)
> 10	1 (0.4)

Notes: *EoL* End-of-life, *ICU* Intensive care unit

#### Self-perceived end-of-life care knowledge of and attitudes towards terminal patients

Critical care nurses reported that ‘I am familiar with the importance of collaboration among end-of-life care team members’ (mean = 4.17; SD = 0.54), ‘I am familiar with the purpose of end-of-life care’ (mean = 4.12; SD = 0.51), and ‘I am familiar with the role and function of an end-of-life care team member’ (mean = 4.12; SD = 0.53) were the most familiar self-perceived aspects of end-of-life care knowledge towards terminal patients, demonstrating higher degree of self-perceived end-of-life care knowledge than other items. The least familiar part of the self-perceived end-of-life care knowledge with the lowest score was ‘I am familiar with the laws relating to end-of-life care’ (mean = 3.42; SD = 0.92), showing that the nurses had lower degree of self-perceived end-of-life care knowledge regarding the laws relating to end-of life care (Table 2). As for the end-of-life care attitudes of critical care nurses towards terminal patients, ‘I believe that providing end-of-life care information to patients and their family is beneficial’ (mean = 4.40; SD = 0.57), ‘I will agree to disconnect the ventilator under legal conditions for a terminal patient’ (mean = 4.38; SD = 0.58), and ‘I believe that high-quality end-of-life care can reduce medical disputes’ (mean = 4.34; SD = 0.62) were the most familiar items, while ‘I have sufficient knowledge to care for patients who have accepted end-of-life care’ was the item with the lowest level of agreement in attitude scores (Table 3).

**Table 2:** Descriptive statistics of critical care nurses’ degree of self-perceived end-of-life (EoL) knowledge towards terminal patients (*N* = 250)

Question	Mean	SD	Order ^*a*^
I am familiar with the importance of collaboration among EoL care team members	4.17	0.54	1
I am familiar with the purpose of EoL care	4.12	0.51	2
I am familiar with the role and the function of an EoL care team member	4.12	0.53	2
I am familiar with handling pain management in EoL care patients	4.09	0.55	4
I am familiar with the clinical symptoms of terminal patients	4.08	0.50	5
I am familiar with the criteria for implementing a DNR	4.08	0.56	5
I am familiar with the definition of EoL	4.06	0.49	7
I am familiar with the importance of communication skills in EoL care	4.05	0.55	8
I am familiar with the ethics and regulations of EoL care	4.03	0.53	9
I am familiar with the needs of the EoL team	4.02	0.55	10
I am familiar with the management of physical discomfort in EoL care	4.01	0.55	11
I am familiar with the mental management of EoL care patients	3.92	0.64	12
I am familiar with the needs of a terminal patient’s family	3.92	0.65	12
I am familiar with the situation when the ventilator is disconnected in EoL care	3.91	0.71	14
I am familiar with the withdrawal of various treatments in EoL care	3.90	0.67	15
I am familiar with considerations regarding the use of sedatives in EoL care	3.90	0.72	15
I am familiar with the appropriate moment to start EoL care	3.87	0.68	17
I am familiar with the content of EoL care	3.85	0.72	18
I am familiar with scope of practice in EoL care	3.82	0.74	19
I am familiar with considerations regarding the use of muscle relaxants in EoL care	3.82	0.75	19
I am familiar with the withholding of various treatments in EoL care	3.73	0.83	21
I am familiar with the administrative support provided in EoL care	3.51	0.91	22
I am familiar with the laws relating to EoL care	3.42	0.92	23

Notes: ^a^Ordered by mean values. *SD* Standard deviation, *DNR* Do not resuscitate

**Table 3:** Descriptive statistics of end-of-life (EoL) care attitudes of critical care nurses towards terminal patients (*N* = 250)

Question item	Mean	SD	Order ^*a*^
I believe that providing EoL care information to patients and their family is beneficial	4.40	0.57	1
I will agree to disconnect the ventilator under legal conditions for a terminal patient	4.38	0.58	2
I believe that high-quality EoL care can reduce medical disputes	4.34	0.62	3
I am willing to sign a DNR for my family member if necessary	4.32	0.65	4
I believe that EoL care should be accepted and understood by the public	4.32	0.67	4
I believe that promoting EoL care can help prevent unnecessary suffering	4.30	0.60	6
I am willing to provide information relevant to EoL care to the terminal patient and their family	4.28	0.59	7
I am willing to give appropriate amounts of morphine to reduce suffering when the blood pressure is unstable in patients who have signed a DNR	4.26	0.67	8
I believe that the concepts of “EoL care” are suitable for non-terminal cancer patients	4.25	0.66	9
I am willing to take time to communicate with a family that has signed a DNR for a terminal patient	4.24	0.58	10
I am willing to take time and effort to satisfy the needs of a patient’s family that has signed a DNR	4.24	0.61	10
I am willing to take time to communicate with a terminal patient that has signed a DNR	4.24	0.62	10
I believe that the process of death of terminal patients is overly prolonged	4.22	0.68	13
I am willing to promote EoL care	4.20	0.63	14
There is sufficient care capability from doctors in the unit for patients that have signed a DNR	4.16	0.68	15
I have sufficient care capability for patients who have signed a DNR	4.14	0.60	16
I have sufficient knowledge to care for patients who have accepted EoL care	4.09	0.66	17

Notes: ^a^Ordered by mean values. *SD* Standard deviation, *DNR* Do not resuscitate

#### Critical care nurses’ perspectives of promoting advance directives

**Fig. 1:**
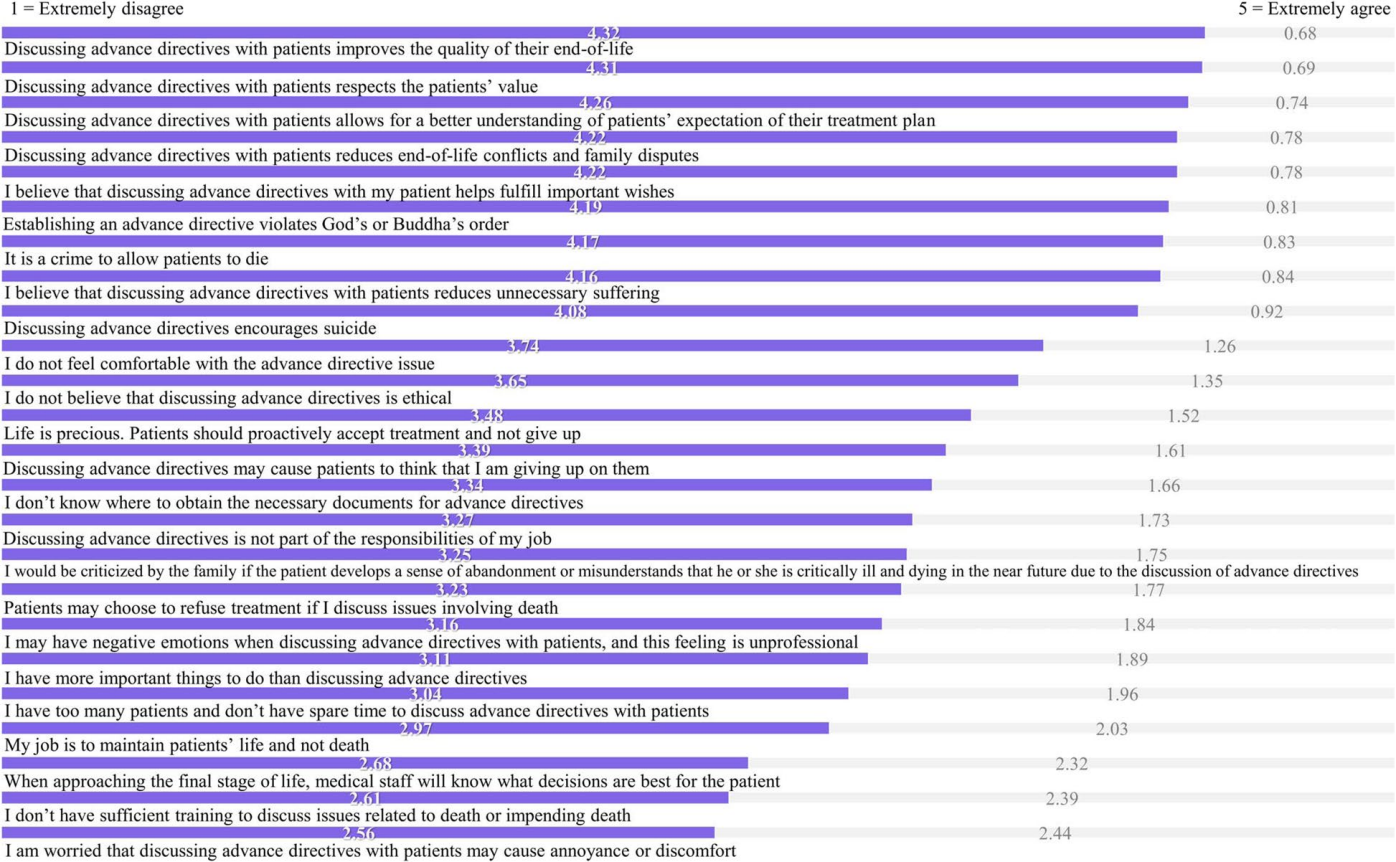
Statements of perspectives of promoting advance directives among critical care nurses. *Note*. Ordered by mean values, the higher the score, the more-positive attitude critical care nurses had towards promoting advance directives. A lower score (1 or 2) indicates that there would be challenges or negative attitudes towards discussing advance directives between critical care nurses and patients

Figure 1 shows that statements with higher agreement of perspectives of promoting advance directives were ‘Discussing advance directives with patients improves the quality of their end-of-life’ (mean = 4.32; SD = 0.53), ‘Discussing advance directives with patients respects patients’ values’ (mean = 4.31; SD = 0.52), and ‘Discussing advance directives with patients allows for a better understanding of patients’ expectations of their treatment plans (mean = 4.26; SD = 0.54)’, and the statement with the lowest agreement was ‘I am worried that discussing advance directives with patients may cause annoyance or discomfort’ (mean = 2.56; SD = 1.01).

#### Factors associated with perspectives of promoting advance directives

The stepwise multiple regression analysis revealed that critical care nurses self-perceived end-of-life care knowledge (*β* = 0.134; *p* = 0.045) and attitudes (*β* = 0.423; *p* < 0.001) towards terminal patients were factors associated with perspectives of promoting advance directives. Nurses with higher self-perceived end-of-life care knowledge and more-positive end-of-life care attitudes had higher motivation to promote advance directives. Also, compared to nurses who worked in medical ICUs, those who worked in cardiac (*β* = -0.234; *p* < 0.001) and respiratory (*β* = -0.135; *p* = 0.024) ICUs had lower motivation to promote advance directives (*F* = 16.943; *p* < 0.001). All VIFs were < 10 which showed that no multicollinearity issue existed in the regression model (Table 4).

**Table 4:** Factors associated with the perspectives of critical care nurses of promoting advance directives (*N* = 250)

Variable	*B*	*SE*	Beta	*p* value	VIF
Constant	50.609	8.359		< 0.001	
Attitude towards EoL care	0.727	0.113	0.423	< 0.001	1.314
Perceived knowledge of EoL care	0.1690	0.084	0.134	0.045	1.322
Type of ICU (Ref: Medical ICU)					
Cardiac	-7.744	1.977	-0.234	< 0.001	1.072
Respiratory	-5.230	2.305	-0.135	0.024	1.076
Regression model^*a*^	*R*^*2*^= 0.225; adjusted *R*^*2*^= 0.211				
	*F*(4,234) = 16.943 (*p* < 0.001)				

Notes: ^a^The stepwise selection was used in the multiple regression model, and non-significant variables were removed from the final model. *SE* Standard error, *VIF* Variance inflation factor, *EoL* end-of-life, *ICU* Intensive care unit

## Discussion

This study assessed critical care nurses' self-perceived knowledge of and attitudes toward end-of-life care, as well as their perspectives of promoting advance directives. Critical care nurses demonstrated high perceived knowledge of end-of-life care on items about the purpose of end-of-life care, the role and function of end-of-life care team members, and the importance of collaboration among end-of-life care team members. This shows that critical care nurses are equipped with fundamental knowledge of end-of-life care and understand that teamwork with other healthcare professionals in the end-of-life care team is important. Other research suggested that interprofessional interventions and interdisciplinary teamwork have the potential to support ICU staff to provide better end-of-life care [12–14]. The least familiar item among critical care nurses was about laws relating to end-of-life care, which referred to the *Hospice Palliative Care Act* in Taiwan [1]. Future studies, interventions, and continuing education can consider putting more efforts in enhancing critical care nurses’ understanding of end-of-life care law.

In line with previous studies that attitudes toward end-of-life in critical care nurses are positive [6, 7, 11], both self-perceived knowledge of and attitudes toward end-of-life care were found to be significant factors of perspectives of promoting advance directives, and future studies can be informed by our findings to develop tailor-made interventions for getting critical care nurses involved and engaged in end-of-life care and decision-making processes [15]. The role of critical care nurses being actively involved in end-of-life care and discussions has been highlighted, and they are essential partners in the end-of-life decision-making process within the end-of-life care team [16]. Increasing nurses’ knowledge and confidence to provide input in end-of-life discussions needs more research attention [17, 18].

Our findings also revealed that critical care nurses realized that discussing advance directives with patients improved their quality of end-of-life, respected their values, and allowed for a better understanding of patients’ expectations of their treatment plans. In addition, critical care nurses were not worried that discussing advance directives with patients might cause annoyance or discomfort. This is dissimilar to a previous study that assessed perspectives of promoting advance directives among nurses working in a hemodialysis room, in which more than 65% of those nurses were worried that discussing advance directives with patients might cause annoyance or discomfort [10]. A possible reason might be that as critical care nurses are frontline care providers who care for critically ill patients, they understand that advance directives would be one of the options to provide better end-of-life care and they have to face many patients with terminal illnesses and dying patients [19, 20]. Also, we identified that compared to nurses who work in medical ICUs, those who work in cardiac and respiratory ICUs have lower motivation to promote advance directives. This is likely because mortality rates in and the severity of diseases are higher in medical ICUs in Taiwan. We suggest adding advance directives-relevant information on laws into the content of continuing education for end-of-life care, particularly for cardiac and respiratory ICU nurses. Educational strategies such as simulated education with role-playing scenarios to introduce advance directives to patients would likely be effective in improving nurses’ end-of-life care [21, 22]. Furthermore, the dignity of patients with palliative needs were also needed to be considered when developing the aforementioned educational strategies for nurses to promote palliative care. As a comprehensive integrated review has summarized a model of dignity that identifying several themes such as family care and support, social justice, reliable health care, which are highly relevant to be embedded in providing nursing care [23].

### Study limitations

The main limitation of this study to be acknowledged is that the survey was only distributed to participants in a major acute-care metropolitan medical center located in northern Taiwan. The nonprobability sampling method used in this study to choose the hospital may influence the external validity of the study, and therefore, the results may not be generalizable to other areas in Taiwan due to representative bias. Future research should enroll more participants that particularly reflect different areas to conduct a multicenter study to compare the results. Nevertheless, this study discovered critical care nurses' perceived knowledge and attitudes toward end-of-life care, as well as their perspectives on promoting advance directives and the associated factors using validated instruments. Findings from this study can inform the design of effective nurse support programs to enhance the promotion of advance directives in intensive care settings.

## Conclusions

Self-perceived knowledge and attitudes toward end-of-life care, and perspectives of promoting advance directives of critical care nurses were assessed. We found that increased levels of perceived knowledge and attitudes toward end-of-life care were associated with the perspectives of promoting advance directives. Nurses who worked in cardiac and respiratory ICUs had less motivation to promote advance directives. This study demonstrated a significant impact of perspectives of promoting advance directives of critical care nurse participants. Given their important contribution to ICU care services, appropriate and meaningful support is required to optimize critical care nurses' involvement in end-of-life care.

## Data Availability

The data that support the findings of this study are available from the corresponding author upon reasonable request.
